# Capturing the pace of extreme diurnal temperature change in climate-health assessment

**DOI:** 10.1016/j.eehl.2026.100262

**Published:** 2026-07-01

**Authors:** Xuankai Ma, Jingzhe Wang

**Affiliations:** a Urumqi Urban Institute of Geotechnical Investigation Surveying and Mapping, Urumqi, 830000, China; b School of Artificial Intelligence, Shenzhen Polytechnic University, Shenzhen, 518055, China

**Climate-health surveillance has made major progress in quantifying heat, cold, and diurnal temperature range (DTR), yet the speed of temperature change remains under-specified in many exposure assessments. We propose extreme diurnal temperature change (EDTC) as a rate-sensitive descriptor of rapid within-day thermal transitions. EDTC should not be treated as a validated universal threshold or as a substitute for DTR. Instead, it offers a complementary way to ask when the same thermal range is delivered over a shorter period, when abrupt cooling or warming occurs, and which populations or places lack the buffering capacity to reduce exposu** **re.**

The 2024 Lancet Countdown shows that climate-related health threats are reaching record-breaking levels worldwide [1]. Mean temperature remains essential for climate-health assessment [2]. However, a large body of epidemiological literature also shows that temperature variability, not only average heat or cold, is associated with mortality [3]. Diurnal temperature range (DTR) is the most established daily measure of within-day thermal amplitude, and multi-country work projects substantial future mortality burdens related to DTR under climate change scenarios [4]. This evidence base should be the starting point for any discussion of extreme diurnal temperature change (EDTC).

## 1 Defining EDTC as a rate-sensitive descriptor

**Fig. 1:**
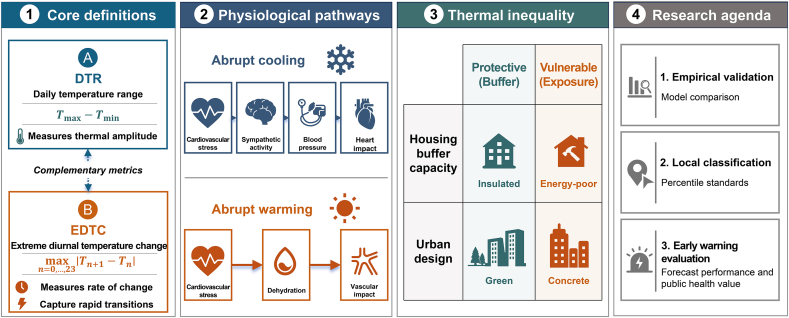
Extreme diurnal temperature change (EDTC) framework for climate-health surveillance.

As illustrated in Fig. 1, we use EDTC to describe the rate of within-day temperature change, expressed in degrees Celsius per hour (°C/h). Operationally, for a day with hourly temperature observations, EDTC can be calculated as:

(1) $$EDTC=\underset{n=0,\ldots ,23}{\max}|T_{n+1}-T_{n}|$$

where *T*_*n*_ is the temperature observed at hour *n*.

This definition differs from DTR, which is calculated as:

(2) $$DTR=T_{\max}-T_{\min}$$

In essence, DTR measures amplitude without specifying how quickly that amplitude occurs.

This distinction is conceptual rather than a claim of superiority. Two days can have the same DTR but different hourly slopes, and therefore different exposure profiles for people moving through outdoor, indoor, and occupational microenvironments. Conversely, a smaller DTR can still contain an abrupt short-lived thermal transition. Existing studies of temperature variability already support the relevance of within-day and hourly scales. Guo et al. [3] found temperature variability to be associated with mortality across 372 locations in 12 countries or regions, and Yang et al. [5] reported an attributable mortality burden from hourly temperature variability across 31 major Chinese cities.

We therefore avoid proposing a universal EDTC threshold. Clinically and atmospherically meaningful thresholds are likely to vary by background climate, season, age structure, housing quality, acclimatization, occupational exposure, and comorbidity profile. Until outcome-specific thresholds are estimated, EDTC should be modeled as a continuous exposure or summarized using locally defined upper-percentile categories. This approach keeps the concept testable without implying that a single global threshold already exists.

## 2 What EDTC can add beyond DTR

DTR remains the benchmark metric for diurnal thermal fluctuation. EDTC adds a different piece of information: the temporal concentration of that fluctuation. DTR asks how far temperature moved during a day; EDTC asks how quickly part of that movement occurred. The added value of EDTC over DTR must be demonstrated empirically in specific datasets and outcomes, for example, by comparing DTR-only, EDTC-only, and combined models for mortality, emergency admissions, asthma exacerbation, or pollutant co-exposure.

The strongest current justification for EDTC is not proven superiority over DTR, but the health relevance already shown for higher-frequency temperature variability. A large multi-country analysis comparing temperature-variability components found that intra-day variability can be independently associated with all-cause, cardiovascular, and respiratory mortality [6]. These findings make rate-sensitive metrics worth testing, especially where exposure changes faster than daily summaries can represent.

## 3 Possible physiological pathways

Rapid thermal transitions can plausibly matter because physiological regulation has latency. Abrupt cooling may increase sympathetic activity, peripheral vasoconstriction, and blood pressure, while abrupt warming may increase cardiovascular strain, dehydration risk, and thermoregulatory demand [4,7]. These pathways are not unique to EDTC, but EDTC can help specify the timing and steepness of the exposure that challenges homeostasis.

Evidence from acute weather-change research supports caution. In a case-crossover study, Rakers et al. [8] found that rapid decreases in ambient temperature and rapid changes in relative humidity and atmospheric pressure were associated with increased ischemic stroke risk, particularly among individuals with higher cardiovascular risk. This does not validate a universal EDTC threshold, but it supports the broader proposition that abrupt meteorological change can be clinically relevant.

## 4 Pollution co-exposure and boundary-layer context

The interaction between rapid temperature change and air pollution should also be framed cautiously. EDTC alone does not determine pollutant concentrations, boundary-layer height, or ozone formation. Pollution episodes depend on emissions, wind, humidity, solar radiation, chemical transformation, aerosol loading, vertical mixing, and regional transport. Severe haze formation in China, for example, has been shown to depend on meteorological conditions and secondary aerosol formation [9], while aerosol-planetary boundary layer feedback can suppress boundary-layer development and intensify haze under polluted conditions [10].

The research opportunity is therefore not to treat EDTC as a standalone proxy for boundary-layer disruption. Instead, EDTC should be paired with boundary-layer height, stability indices, wind speed, humidity, PM_2.5_, ozone, and activity-pattern data. The key question is whether rapid cooling or warming episodes identify short windows when physiological stress and pollutant exposure co-occur. This is a testable hypothesis, not a settled causal pathway.

## 5 Thermal inequity

EDTC also draws attention to unequal buffering capacity. Exposure to a rapid outdoor temperature transition is mediated by housing, work, transport, clothing, energy access, and warning systems. Building envelopes, insulation, ventilation, and climate control can reduce the speed and magnitude of indoor exposure changes, whereas poorly insulated housing can transmit outdoor volatility indoors [11]. Studies from the smart wellness housing survey in Japan show that lower indoor temperatures and unstable indoor temperatures are associated with higher home blood pressure and blood-pressure variability [12].

This point does not conflict with concerns about heat-retaining urban materials. The two mechanisms operate at different scales. Indoor insulation can protect individuals by stabilizing rooms. At the neighborhood scale, impervious surfaces, sparse vegetation, street geometry, and anthropogenic heat can alter outdoor heat storage, nighttime cooling, and microclimate variability. A thermally resilient city must therefore combine building-level buffering with outdoor urban design that reduces exposure to abrupt and uneven thermal transitions [13].

## 6 Conclusion

EDTC should be developed as a research agenda before it is used as a policy threshold. The next step is not for institutions to adopt a fixed thermal velocity threshold, but for epidemiological and atmospheric studies to test whether rate-sensitive metrics improve risk assessment beyond established variables. Priority analyses should compare EDTC, DTR, daily mean temperature, day-to-day temperature change, humidity, and pollution variables in the same models; estimate lag structures and susceptible subgroups; and evaluate whether local percentile-based EDTC categories improve early-warning performance.

This reframing preserves the central insight that the pace of temperature change may matter for health, while aligning the claim with the available evidence. In a warming and increasingly variable climate, monitoring should capture not only how hot or cold a day becomes, but also how quickly people, buildings, and urban atmospheres are forced to adjust.

## CRediT authorship contribution statement

**Xuankai Ma:** Conceptualization, Formal analysis, Investigation, Methodology, Resources, Validation, Visualization, Writing – original draft. **Jingzhe Wang:** Conceptualization, Formal analysis, Funding acquisition, Investigation, Methodology, Resources, Validation, Visualization, Writing – original draft.

## Declaration of competing interest

The authors declare no competing interests.
